# Open Dialogue: A case study on the influence of sharing or withholding reflections during a network meeting

**DOI:** 10.3389/fpsyg.2022.1028757

**Published:** 2022-12-01

**Authors:** Albert van Dieren, Corine Clavero

**Affiliations:** ^1^Ede Christian University of Applied Sciences, Ede, Netherlands; ^2^Christian University of Applied Sciences, Ede, Netherlands

**Keywords:** network meeting, inner outer dialogues, dialogism, reflections, significant and meaningful moments

## Abstract

In Open Dialogue, sharing of reflections by professionals constitutes an important contribution to promoting a polyphonic dialogue between participants. In the inner dialogue, past and future influence the present moment. In this study, we explore the influence of sharing or withholding reflections by professionals on the interplay between inner and outer dialogue. A case study was used with a multi-perspective methodology, which combined video recordings of a network meeting and interviews by using video-stimulated recall with the clients separately, and social workers together afterward. We found that the sharing of reflections by professionals stimulates the inner dialogue and creates an opening for sharing these in the outer dialogue. In addition, we observed that when reflections are withheld, the client's inner dialogue still continues, but their inner dialogue was not shared in the outer dialogue.

## Introduction

Since January 2015, a transformation in the social domain in the Netherlands has taken place with the introduction of new legislation contained in the Participation Act and the Youth Care and Chronic Care Act (Kelders et al., 2016). This change has affected all aspects of the social domain such as the cutback of financial flows in the healthcare system, but also a shift in the organization of welfare and care from the Dutch government to local authorities and citizens. Self-reliance is promoted, together with informal caregiving from family carers or by volunteers or other important members of the social network (Dekker and Van Dieren, 2016).

This transformation requires social workers to work actively with clients and members of their social network from the onset of care or support. One of the key principles of social work is that the professional actions of the social worker should involve how to coach the client to develop themself in relation to their social environment and within the socio-cultural context (Van der Mei and Luttik, 2018). This is in line with the knowledge and research agenda of social work in the Netherlands, which promotes social network meetings where members of the informal network will actively participate in the meeting. In recent years, knowledge has been developed about social network approaches and social network reinforcement. But researchers acknowledge that more practice-based knowledge is needed on how to apply informal network approaches in daily life practice (Hooghiemstra et al., 2020).

Recent research work shows that many social workers are reluctant to do so. Most of them confirm the importance of this way of work, but in everyday practice, it is not often operationalised. Previous research shows that many social workers experience:

- Inability to work with the client and their social network because most treatments are individually organized
- Finding attunement care or support is a relational quest and this demands relational and dialogical skills from the social worker
- Feeling insecure due to a lack of knowledge, tools, and training (Van Regenmortel and Lemmens, 2012)
- Organizations promote working with social networks but do not have a vision of how to do so (Dekker and Van Dieren, 2020, 2021).

Our assumption is that Open Dialogue can help professionals conduct network meetings to create space for the participants' worries and needs. It creates a place where all participants will be heard and be together to find new constructive ways of dealing with concerns that can emerge (Seikkula and Arnkil, 2006, 2017).

Dialogue is seen as a way to have a conversation about problems or worries between the client(s) and their social network members in addition to finding a way to acquire more agency in their lives. It is a mutual or shared inquiry of listening (Anderson, 2007), questioning, and reflecting on the problem, to find new ways to go forward. The social worker will respectfully respond to the utterances of the participants and will share their own reflections and invite the others to react—in order to avoid a one-sided context (Olson et al., 2012, 2014; Seikkula and Arnkil, 2017).

A network meeting has three functions, according to Alanen (1997): (1) to gather information about the experienced problem, (2) to create a treatment plan, and (3) to generate dialogue. In the dialogue, the focus will be on strengthening the client's adult side (Seikkula, 2002) and on having a discussion that views the client's behavior as meaningful (Alanen, 1997; Anderson, 2007; Olson et al., 2014; Seikkula and Arnkil, 2017). Dialogue is a relational and collaborative activity (Anderson, 2007). In the dialogical process, different voices emerge. First, there is the horizontal polyphony or outer voices. These are the words spoken by the participants. But there is also a vertical polyphony or inner dialogue present in the dialogue. These are the thoughts and feelings each person has during the meeting that are not yet spoken out loud. In generating dialogue the aim is to create space for “the not yet said” (Anderson and Goolishian, 1988; Rober, 2002). The dialogue comprises not only verbal utterances but includes all responses including (signs of) empathy, compassion, emotions, and bodily changes connected to the process of meaning-making in social interaction (Bertrando and Arcelloni, 2014; Kykyri et al., 2017). All voices or perspectives are equally important and it is important to respond to these as professionals (Seikkula and Arnkil, 2017; Ong and Buus, 2021). In the dialogue, professionals are not seeking agreement about the problem but they invite as many voices or perspectives as possible so that new meanings can emerge (Anderson, 2007; Rober, 2012; Lidbom et al., 2014). From this point of view, you could say that all these utterances occur in the present moment. But in the dialogue, the past resonates, and in the answers, the future can emerge. Thus, in the dialogue, the future can serve as an inspiring viewpoint for creating new meanings, actions, and understandings (Boe et al., 2015; Seikkula and Arnkil, 2017).

During a network meeting, professionals create an opportunity for clients and network members to share reflections while others are listening. In doing so the professionals will look at and talk to each other and not to the family or network about what they have heard during the conversation. During this reflecting process, the professionals will share ideas, images, and metaphors that came to mind during the conversation with the client and family. Andersen (1995) formulated some guidelines regarding procedures for guiding the reflective process. Reflections should be based on what has been said or expressed during the conversation. It is important that the shared reflections are not statements, opinions, or assertions of meaning, but are formulated as ideas or suggestions. Statements and opinions can be easily heard as criticism. Another rule is that the professionals will avoid negative reflections. The professionals are encouraged to use ordinary language, and professional jargon should be avoided (Andersen, 1992, 1995; Olson et al., 2014). Seikkula and Arnkil (2017) state that when professionals are reflecting, others will be present in their inner dialogue by listening. By inviting them to respond to the reflection, a space is created so that they can share their inner dialogues. The goal is to create space for new conversations where new meanings can arise during the network meeting and the participants can find ways to move forward. Sharing reflections may create space for “the not yet said” (Anderson and Goolishian, 1988; Rober, 2002).

In a network meeting, the concepts of sharing reflections, inner and outer dialogue, and time influence the dialogical process.

This study aims to find answers to the following research question: What influence has the sharing or withholding of reflections by the social workers on the inner and outer dialogue in a network meeting?

## Methods

Our assumptions led to a research project in a Youth Care organization on how social workers work together with children/teenagers, parents, and other social network members. In this action-oriented research, we work with professionals who are open to changing the way they work and want to work actively together with the social network (Dekker and Van Dieren, 2020, 2021). In November 2020, the social workers received basic training in the dialogical approach in network meetings. During and after this training, we studied how the dialogical approach can help children, teenagers, parents, social network members, and professionals to find attunement regarding social care or support. As a result of this training, two social workers recorded a network meeting which we used for the case study. One of the social workers is the second author of this article.

In the case study, we explored whether sharing reflections or withholding reflections by the social workers influenced the inner and outer dialogue of all involved.

Inspired by the research work of Rober et al. (2008) and Lidbom et al. (2014), we wanted to explore the dialogical process in a case study by seeing how sharing reflections influenced all those who are involved in the network meeting. This is a qualitative case study of a father and son, who received care after the mother passed away.

The son had entered the local youth care a couple of years earlier, at the age of 15, due to an autistic spectrum disorder. He was referred to a farm care programme one weekend a month to relieve the family and help him learn to deal with his problems. When social worker 1 met the family for the first time, it became clear that more help was needed because of the son's angry outbursts and depressive symptoms. At first, the conversations were only with the son, but early in the process, the social workers decided that it might be helpful to have meetings with the father and the son together.

A year later we recorded one network meeting with the father and the son and two social workers. The actual network meeting was organized by the youth care organization and took place at the father and son's home. The network meeting was recorded and lasted 1 h and 15 min. Before this meeting, social workers had several meetings with the son alone and met four times with the father over a period of 11 months. After recording the network meeting the participants were interviewed afterward by the first author, using the video-stimulated recall method (Rober et al., 2008; Nguyen et al., 2013). To analyse the content of the inner and outer dialogues, the reflections, and the interplay between them, we made use of the dialogical concept of Sullivan for qualitative data analyses. This approach provides tools to analyse subjectivity in qualitative data. “Subjectivity is theorized as changing and responsive to others” (Sullivan, 2012, p. 1). In particular, we made use of the concept of key moments which contain utterances of significance, reflection, and relational impact (Sullivan, 2012, p. 21–23).

During the network meeting, three main topics were discussed. The three topics were the evaluation of how the son was behaving socially, the relationship between the son and the father, and the ending of the support relationship with one of the social workers. The chosen fragments appear in the second part of the conversation.

The first stage of the research was recording the network meeting. The second stage was to watch the recording with the son and the father separately the day after the recording. We asked them to stop the recording at significant moments and to answer the question, “what was on your mind at that moment?” No other questions were prepared for these interviews. The answers were recorded on video. The procedure was repeated with the two social workers together 2 days after the recording, using the same method and asking the same question. This meeting was also video-recorded.

The third stage was to transcribe all the recordings. The fourth stage was to select recording fragments. Only those fragments were selected where all participants stopped the recording and shared their inner thoughts and feelings. Thirteen selections were made and all four participants stopped the recording around the same time and stated these moments to be significant. We did not obtain the exact time of pausing for all four participants. The video was paused with a couple of seconds difference between each participant. It was often shared that they were looking for the right moment to pause and we observed that they all talked about the same topic. Thus, we came close enough to be able to put the selections together. During the next stage (stage five), from those selections, we placed the outer and inner dialogues of all involved in the meeting in the correct position according to the pause they had made in the video recording. In the sixth stage, we looked for the presence and/or withholding of shared reflections in those selected recordings, and we examined what happened in the inner dialogue and what followed after that in the outer dialogue.

From those selected recordings, we chose one fragment that shows the presence of shared reflections and one fragment that shows the absence of shared reflections to build our analysis upon. The first was chosen by the first author as meaningful because of the rich content of the inner dialogue of all participants while the sharing of reflections was absent. The second fragment was selected because the father and son called this a powerful and meaningful moment after a reflection was shared.

### Chosen fragments and analysis

#### The first fragment: Withholding of sharing reflections

The topic of this fragment was evaluating changes in the relationship between father and son (Table 1).

**Table 1:** Analysis matrix absence of reflections.

**Social worker 1**			**Social worker 2**			**Father**			**Son**		
**Outer dialogue**	**Directed at**	**Inner dialogue**	**Outer diaogue**	**Directed at**	**Inner dialogue**	**Outer dialogue**	**Directed at**	**Inner dialogue**	**Outer dialogue**	**Directed to**	**Inner dialogue**
						But what has changed is that – Yes, somehow I notice that he has started to take me a lot into account, in the sense of hey maybe I should stop asking questions in the evening for all kinds of conversations.	All, but especially to the son.				
								Yes, this is where I lose my son somewhere. Every time I think of hey– Well, that's why I was looking for words in the beginning, because before you know it, you'll hit the wrong botton with him while you want to give him a compliment. It just hurt very much. I'll close myself off to you. That is not the case. I found that a really painful moment.	Doesn't necessarily have to do with taken into an account but more with I've given you a shitload of information and now I'm just closing myself off to you.	Son to Father	
		That continues to touch me because somewhere I see how hard father is trying and how much baggage he has and he is also is vulnerable and really tries to be a father. While I can also understand the son very well of all the pain that is in him that he finds this so difficult. And we've talked about that several times.				Ok. I can do that too. I don't think that's true, but if you experience it that way, you may.	Father to Son				
			Because what makes you say that? I'm curious?	SW 2 to Son							I was like yes it's fine to park it. SW2 says why are you saying this? I was like yes, I'm really attacking my dad here. I shouldn't have been so attacking. Why did I say this? I actually started to think about that whole choice of why did I say this. Why am I literally trying to hurt my father here? So when SW 1 said do you want to park this? Then I was like– Oh let's park this. Because my father is going to play a role in the rest of my life **and it might also be useful to discuss things like that with my father. Only I have no idea how**. Then I thought I must remember this, I still have to talk about this.
Is it ok to park it for a while?	SW 1 to SW 2	Yes, because you're asking a very good question, right? But I had been here with them before and talked about it and I was thinking is the son going to be central again and then I see father disappear into the background.						And there were all kinds of process interventions here. Well I have just tried to give my son a compliment, but he has rejected it, saying I close myself off to you anyway. Well and that's why I'm now also like how are we going to put this into words? How should			
								I do this? And my son who picks that up continuously when I am completely burned out. Yes, then I feel so inadequate.			
			Yes, you may	SW 2 to SW 1	**Well, for me it wasn't when I go to the son that I didn't father– But you start somewhere. So that same question goes up I go back to father**. But you know, I also felt that you want to stop him very consciously so then I'm not going there either– You have that pre-knowledge.						
Because otherwise we go there – I'm very curious what dad– Is that ok for you?	SW 1 to SW 2	Then it will be fifteen minutes longer at least. And I didn't want that. So I thought I just want to stay with dad now. *But I did want to check that with you kind of*.			kind of…						

Red, past; Green, present; Blue, future.

Italics, inner dialogue during primary conversation.

Bold, inner dialogue during reflection in the reconstruction conversation.

#### Outer dialogue

The outer dialogue concerns how the father wanted to compliment the son on how he had changed his behavior. The son's response was offensive to the parent. Social worker 2 (male) asked the son the reason for this response and social worker 1 (female) requested that the question be parked.

#### Inner dialogue

Social worker 2 asked the question to the son, and social worker 1 wanted to park the question. In the inner dialogue, social worker 1 was afraid that the question would lead to a repetition of the interaction she had experienced in previous meetings with the father and son. Social worker 2 heard the request of social worker 1 to park the question and felt that social worker 1 probably had good reasons for her request, so he agreed.

The inner dialogue of the parent during this fragment was that he wanted to give a compliment to his son. He wanted to tread carefully so that the teenager could accept the compliment. The son's reaction evoked painful feelings of incompetence in the father. In the inner dialogue, the son was wondering why he had this reaction toward his father because the son realized that the relationship with his father will continue one way or another whereas the relationship with the social workers will come to an end.

### The interplay between the participants' inner and outer dialogues

The topic during this fragment concerned the progress the son has made in his behavior over the last 11 months. In the inner dialogue, the father was carefully looking for words to express the improvement. In the outer dialogue, the father said “*Yes, somehow I notice that he has started to take me into consideration a lot in the sense of - hey maybe I should stop asking questions in the evening in all kinds of conversations”*. The son reacted “*Doesn't necessarily have to do with taking you into consideration, but more with I gave you a shitload of information and now I'm just close myself off to you”*.

In the outer dialogue, social worker 2 asked “*What makes you say that? I'm just curious?”* Social worker 1 asked social worker 2 “*if it was okay to leave the question for the moment and not go in this direction”*. Social worker two answered, “*That is fine*”.

In the inner dialogues, completely different meanings were experienced. Because of earlier experiences of outbursts from his son, the father was careful about what and how to say things and how to give a compliment. In the recall interview, the son explained his reaction toward his father. The question by social worker 2 made the son realize that this way of reacting was “*very offensive”* toward his father. The son was glad that social worker 1 wanted to park the question. In the inner dialogue, the father experienced the reaction of his son as: “*I feel like I'm falling short”*. In the outer dialogue, the father expressed that he doubted if the reaction was genuine, but respected his son's feelings. The father questioned the son's reaction because in everyday life the son's behavior shows something different. After watching this fragment, the son started to reflect on his reaction and started to reconstruct ways to get along with his father in the future. “*I realised I have to deal with this because my father will play a role in my life and SW 1 will be gone from my life”*.

In the inner dialogue of social worker 1, the interaction between father and son was experienced differently compared to social worker 2. Social worker 1 did not want to discuss the reasons for the son's reaction toward his father. The reasons not to address the subject were due to earlier experiences with the pair and the hope of giving space to the father to express himself.

In the inner dialogue, social worker 2 was not aware of that and experienced the request (to park the question) as inappropriate, believing that social worker 1 thought that “*if I asked the son this question, it wouldn't invite father to respond. But I felt you wanted to stop him”*. Social worker 2 felt that social worker 1 wanted to stop this interaction and agreed to park the question for the moment because *he felt that she had prior knowledge of the situation*.

Interestingly, it may appear that the dialogical space was closed at the request of social worker 1. But in the inner dialogue between father and son, many things happened. This also makes it clear how important it is to be sensitive toward clients' expressions and to invite them to explore the connected inner dialogue. It also clarifies the fact that earlier experiences can make a social worker hesitant to enter the dialogue on topics that had been discussed in the past (Olson et al., 2012; Seikkula and Arnkil, 2017). Social worker 2 was curious why the son reacted this way and wanted to know how it was experienced by the father as well. In this fragment, the attunement between the two social workers was disturbed, which led to a closure of the outer dialogue.

#### The second fragment: Withholding of sharing reflections

The topic during this fragment was the relationship between the teenager and the parent (Table 2).

**Table 2:** Analysis matrix sharing of reflections.

**Social worker 1**			**Social worker 2**			**Father**			**Son**		
**Outer dialogue**	**Directed at**	**Inner dialogue**	**Outer dialogue**	**Directed at**	**Inner dialogue**	**Outer dialogue**	**Directed at**	**Inner dialogue**	**Outer dialogue**	**Directed to**	**Inner dialogue**
									English humor is the most fun humor, so sometimes I can take it and sometimes I can't. And if I don't handle it then it's very annoying.	Son to SW 1	
Because it also touches you again, do I matter? If I say that so correctly. And if your father sometimes tries to say something positive with some English humor, you feel that tone and that touches you. And that happened again somewhere.	SW 1 to Son							Well he points out so I can handle it. Well that makes it difficult for me to understand it again. Sometimes he can and the other time it can't handle. Because I noticed by myself again the– Yes, you want to defend yourself.			
									What was the trigger, yes, but that's who I am. Easiest excuse ever. Even if you shoot me, I won't get rid of it.	Son to SW 1	
I hear your father searching for where is it that things go wrong because I try to do it right and sometimes I don't quite get it. But I don't know if that's true?	SW to Father & Son										
						In my experience, but maybe I am wrong, but can you basically handle it. We also have fun together about crazy things or whatever.	Father to Son				
						In fact, in my experience, a chip off the old block, you also make those sharp comments.					
									The difference is between sometimes and always.	Son to Father	
I have to think a little bit about us	SW 1 to SW 2	I really was searching									
			Yes, for me it's why can we do that, because you are his father and you are his son, because you want to be seen by your father and feel that you are the most important to him.	SW to Father & Son	But there are other things going on here, yes, I can feel them in relation to my own father.						I thought it was so brave of SW 2 that he gave me a tap on my shoulder at the right time.
									Yes, because with friends I can be very sarcastic.		
		I was really happy with that.	And the moment that you have not felt that with your father at times, that comes so deep inside because you are his son. And that has been given a place somewhere I think and that's what it touches on.	SW 2 to Son				This is what I really liked about SW 2, this summary. **That rang a bell for me**.			SW 2 mentions the core of the problem here, **which I really liked**
									**That is it**.	Son to all	

Red, past; Green, present; Blue, future.

Italics, inner dialogue during primaire conversation.

Bold, inner dialogue during reflection in the reconstruction conversation.

#### Outer dialogue

The outer dialogue is about how humor is used in the father-son relationship and how social worker 2 mentioned that the reason why jokes do not always come across well is that the relationship between a father and son is different from a relationship with a friend.

#### Inner dialogue

Social worker 2 felt that other things are going on during the conversation beyond just not understanding the humor between father and son. In the inner dialogue of social worker 2, it becomes clear that “*he can feel it in his relationship with his own father”*. He then introduces this as a reflection after social worker 1 states to him that she is thinking of their relationship.

The father's inner dialogue shows that this shone a different light on the situation. In the inner dialogue, he states that “*he liked the summary of social worker 2* and *that rang a bell”* for him.

While social worker 2 was sharing his reflection with the father and the son, he tapped the son on the shoulder. Earlier in the conversation, the son had clearly said he did not want to be touched by his father. In the son's inner dialogue he shares that “*he found it so brave that Social Worker 2 tapped him on the shoulder at the right time* and *said that the core of the problem is expressed here”*. It is the son who in the outer dialogue says: “*This is it”*.

During the meeting, multiple inner voices and bodily expressions can be evoked. Social workers can actively use their inner voices and experiences and share these in dialogue with each other (Rober, 2005).

### The interplay between the participants' inner and outer dialogues

The topic during this fragment concerned the relationship between the son and the father. The subject was about how they use humor in their relationship. It was unclear to them why they sometimes misunderstood each other while joking. The son states that he does not encounter the same misunderstandings with his friends.

Both social workers experienced that this was an important moment. Social worker 1 tried first with an explanation of what might have happened, which lead to a statement by the son that this was simply the way he is.

Then social worker 1 turned to social worker 2 and tried to engage with him, with their own relationship as a starting point. In her inner dialogue, she shared that she was searching for how best to continue. However, this opened up the opportunity for social worker 2 to share his reflections on the difference in relationships between fathers and sons and that the son wanted to be acknowledged by his father and know he is important to him. This emerged from his reflection on his relationship with his own father.

Being able to share this reflection with the father and the son, another voice was added to the conversation, which sheds a different light on the previous discussion on how well both are able to handle certain jokes. This lead in their inner dialogue to a reconstruction of the situation, which the son also makes clear in the outer dialogue. It helped to ease the discussion between the father and son. It had the same effect on the father, as he states in his inner dialogue that it rang a bell for him at that moment.

In this fragment, the social workers were able to find attunement between each other which led to an opening for social worker 2 to share his reflections. Thus another voice was added to the conversation which brought a new perspective to the situation (Rober et al., 2008). Adding a new perspective opened an opportunity to create a new meaning between father and son. This becomes clear in the inner dialogue and in the outer dialogue. It is apparent that the sharing of reflections gives meaning to the conversation and creates an opening for the dialogue to continue (Seikkula and Trimble, 2005; Seikkula and Arnkil, 2006).

In addition to what happens in the outer dialogue, a lot happened in the non-verbal communication between social worker 2 and the son. Earlier in the conversation, the son shared that he does not want to be touched. In this fragment, social worker 2 taps the son on the shoulder. In the son's inner dialogue, he saw this as a significant moment.

## Discussion

In this case study, we found that sharing and withholding reflections can influence the inner and outer dialogues of the clients. This finding opens the door to more research on this topic for a broader view of the influence of sharing and withholding reflections and how social workers can find attunement in network meetings. Since it is the task of social workers to conduct more network meetings, we assume that if they are trained in this way of conducting the meetings they will feel more competent in this role.

In this case study, the network meeting was led by two social workers. We found that it was of benefit to the network meeting to have two social workers participate. While one social worker was more actively involved in the conversation, it allowed the other social worker to listen. In this way, he could let the conversation resonate in his inner dialogue and share this with the father and the son. Through the two fragments, we found out that it is important that the social workers also find attunement between each other. This observation could form an interesting premise for more research on how social workers attune to each other in the present moment.

We also want to point out how the method of stimulation recall could be useful in social work. For example, in the fragment of sharing of reflections, it is worth mentioning that social worker 2 not only shared a reflection but also added a personal voice to the dialogue. Besides the personal voice, he tapped the shoulder of the son, while the son earlier shared that he did not want to be touched. This could be interpreted as crossing a boundary. Nevertheless, the son found it brave of social worker 2, which became clear during the stimulated recall. During the network meeting itself, the son stated this as the core of the problem by saying: “*This is it”*. Even though social worker 2 overstepped a boundary for proper professional conduct, he stepped into the dialogue-friendly social space.

It has been remarkable to have observed the reconstructed meaning by reviewing the network conversation with the clients. Using the method of video-stimulated recall brings up the question of whether and how this may be used more frequently after network meetings. The thoughts and feelings expressed in the recorded session belong to that of the present moment. Reviewing the recording from the previous day, and reflecting on the thoughts and feelings from that moment, led to new ideas and emotions at the present moment of watching the video. During that process, a new meaning was reconstructed, which enriched the relationship between the father and the son. For example, the son stated: “*I realised I have to deal with this, because my father will play a role in my life and social worker 1 will be gone from my life”*.

This happened while watching the video fragment where social worker 1 had asked social worker 2 to park the question in the video. Without the review, the son's inner dialogue would have remained unknown and there would have been no possibility to construct a new meaning. This raises the question of if social worker 1 had not asked social worker 2 to park the question, whether the son would have come to the same conclusion or not. Yet, only because of watching the video the next day, the son was able to make this statement.

More research is needed on how clients can benefit from this method and how this could be used in the process of conducting network conversations. And if this method is used, does it promote or undermine the basic principles of dialogism (Anderson, 2007; Anderson and Gehart, 2007), such as transparency and creating polyphony in network meetings?

This case study shows that the sharing and withholding of reflection by professionals in a network meeting has an influence on the inner and outer dialogues of all participants.

The sharing of reflections by the social workers led to clarity in the relationship between the father and the son. It showed the difference in comparison to the relationship with friends. In the inner dialogue between the father and the son, we see that this is a significant moment for them. It is the son who shares in the outer dialogue how the reflection resonates with him.

The outcome was that there was a better understanding between father and son, which strengthened their relationship and achieved attunement in the conversation.

In this study, we have seen the importance of sharing reflections in a network meeting, which contributes to giving an opening in the outer dialogue to share one's inner dialogue.

## Data availability statement

The original contributions presented in the study are included in the article/Supplementary material, further inquiries can be directed to the corresponding author/s.

## Ethics statement

Ethical review and approval was not required for the study on human participants in accordance with the local legislation and institutional requirements. Written informed consent to participate in this study was provided by the participants' legal guardian/next of kin. Written informed consent was obtained from the minor(s)' legal guardian/next of kin for the publication of any potentially identifiable images or data included in this article.

## Author contributions

All authors listed have made a substantial, direct, and intellectual contribution to the work and approved it for publication.

## Funding

Ministerie van Volksgezondheid, Welzijn en Sport (VWS) grant number 332041.

## Conflict of interest

The authors declare that the research was conducted in the absence of any commercial or financial relationships that could be construed as a potential conflict of interest.

## Publisher's note

All claims expressed in this article are solely those of the authors and do not necessarily represent those of their affiliated organizations, or those of the publisher, the editors and the reviewers. Any product that may be evaluated in this article, or claim that may be made by its manufacturer, is not guaranteed or endorsed by the publisher.
